# Electrospun PEO/PEG fibers as potential flexible phase change materials for thermal energy regulation

**DOI:** 10.1002/EXP.20230016

**Published:** 2023-09-18

**Authors:** Xiang Yun Debbie Soo, Sze Yu Tan, Augustine Kok Heng Cheong, Jianwei Xu, Zhiyuan Liu, Xian Jun Loh, Qiang Zhu

**Affiliations:** ^1^ Institute of Materials Research and Engineering (IMRE) Agency for Science, Technology and Research (A*STAR) Innovis Singapore; ^2^ Institute of Sustainability for Chemicals Energy and Environment (ISCE2) Agency for Science, Technology and Research (A*STAR) Jurong Island Singapore; ^3^ Department of Chemistry National University of Singapore Singapore Singapore; ^4^ Shenzhen Institute of Advanced Technology (SIAT) Chinese Academy of Sciences (CAS) Shenzhen People's Republic of China; ^5^ Department of Material Science and Engineering National University of Singapore Singapore Singapore; ^6^ School of Chemistry Chemical Engineering and Biotechnology Nanyang Technological University Singapore Singapore

**Keywords:** confinement effect, electrospun, fibrous mat, polyethylene glycol, polyethylene oxide, thermal management

## Abstract

Polyethylene glycol (PEG) is widely used as phase change materials (PCM) due to their versatile working temperature and high latent heat. However, the low molecular weight of PEG prevents from the formation of flexible microfibers, and the common leakage problem associated with solid–liquid PCM further hinders their applications in various fields. To address these challenges, polyethylene oxide (PEO) is incorporated as the supporting matrix for PEG, leading to a successful electrospinning of fibrous mats. Due to the similar chemical nature of both PEG and PEO, the blended composites show great compatibility and produce uniform electrospun fibers. The thermal properties of these fibers are characterized by DSC and TGA, and supercooling for the PEG(1050) component is effectively reduced by 75–85%. The morphology changes before and after leakage test are analyzed by SEM. Tensile and DMA tests show that the presence of PEG(1050) component contributes to plasticization effect, improving mechanical and thermomechanical strength. The ratio of PEO(600K):PEG(1050) at 7:3 affords the optimal performance with good chemical and form‐stability, least shrinkage, and uniformity. These fibrous mats have potential applications in areas of food packaging, flexible wearable devices, or textiles to aid in thermal regulation.

## Graphical abstract

Thermal regulating fabrics are highly applicable in various industries such as bandages, wearable devices, clothing, or facemasks. In this work, PEO is electrospun with PEG as the PCM and plasticizer component, which show great compatibility and produce uniform fibers. Presence of PEG significantly improve the fiber's mechanical properties, and supercooling for PEG component effectively reduce by 75–85%, improving the fiber's overall functionality.

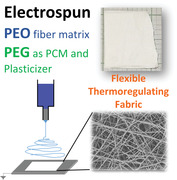

## 1 INTRODUCTION

The rising global temperatures have led to a growing interest in thermal management with PCMs being identified as a promising solution in many industries including healthcare, electronics, logistics, construction, textiles etc.^[^ ^1^, ^2^, ^3^ ^]^ The key advantage of using PCM is their broad working temperature range together with high energy storage capacity, making them suitable for cooling/heating or energy storage purpose in many fields. Nevertheless, a common problem for solid–liquid PCM is leakage during the phase transition,^[^ ^4^, ^5^, ^6^ ^]^ which has been addressed through a few solutions such as macro,^[^ ^7^ ^]^ micro^[^ ^8^, ^9^ ^]^ and nano^[^ ^10^, ^11^ ^]^ encapsulation, or fabrication of form‐stable PCMs (FSPCMs)^[^ ^12^, ^13^, ^14^, ^15^, ^16^ ^]^ via polymeric blending,^[^ ^17^ ^]^ vacuum impregnation,^[^ ^12^ ^]^ physical entanglement,^[^ ^18^ ^]^ or chemical crosslinking.^[^ ^19^ ^]^ While micro and nano encapsulation methods may face challenges of the feasibility of large scale production and high costs, macro encapsulation is a suitable method for large sized applications. FSPCMs work based on the concept of incorporating PCM into a supporting matrix to prevent leakage of PCM during the phase transition, although some leaching may occur in the long run.^[^ ^20^ ^]^ Compared to micro or nano encapsulation, the fabrication methods of FSPCMs are simpler and it can achieve higher yields with comparable latent heat, which is critical for thermal energy absorbance and release, unlike thermoelectric materials that convert heat into electric energy.^[^ ^21^, ^22^, ^23^, ^24^ ^]^

In search of a flexible material for textiles, wearable devices, and thermal bandages, different types of flexible FSPCMs have been developed in the form of membranes/films/sheets, shape memory polymers, or fibers. They can be fabricated via different methods such as melt blending of PCM‐polymer mixture,^[^ ^25^, ^26^ ^]^ PCM diffused or impregnated within a 3D flexible polymer matrix,^[^ ^13^, ^27^ ^]^ melt‐spun^[^ ^28^ ^]^ or electrospun^[^ ^29^, ^30^ ^]^ into micro or nano PCM fibers etc. Of these materials, fibers are in particularly interesting in cooling applications given the porosity and large surface area of the material which allow for efficient heat exchange. Electrospinning is a highly versatile method used to fabricate micro or nano fibers from different types high molecular weight polymers such as polyacrylonitrile (PAN),^[^ ^31^ ^]^ poly(vinyl alcohol),^[^ ^32^ ^]^ polylactic acid (PLA),^[^ ^33^, ^34^, ^35^ ^]^ cellulose^[^ ^36^ ^]^ etc. By changing its parameters (voltage, solution viscosity, solution feed rate, needle height etc.), the fibers’ morphology, porosity, and thickness can be tuned to generate a highly flexible material. However, fibers spun by only one material are generally stiffer due to stronger interaction, such as via hydrogen bonding, within the polymer matrix. Hence, plasticizers are added to reduce the inter‐chain interaction and increase film flexibility.^[^ ^37^ ^]^ They are usually low molecular weight, non‐volatile compounds, and a compatible plasticizer should be able to tune the fiber's physical properties without affecting the fiber's chemical nature.^[^ ^38^ ^]^ PCMs are also often incorporated into the fibers in the form of microcapsules,^[^ ^39^ ^]^ direct mixing^[^ ^36^ ^]^ with the polymer solution and electrospun as fibers, or via a post fabrication step of crosslinking^[^ ^29^, ^40^ ^]^ with the components to generate a thermo‐regulating fabric.

PEG is a commonly used PCM owing to its high solidification enthalpy and wide working temperature, non‐toxic nature, good biocompatibility, and chemical and thermal stability.^[^ ^41^, ^42^, ^43^, ^44^, ^45^, ^46^, ^47^, ^48^, ^49^ ^]^ It is frequently incorporated as the PCM component in electrospinning polymer solutions, and electrospun as PCM fibers. PEG is also commonly used as a plasticizer^[^ ^50^, ^51^, ^52^ ^]^ given its high compatibility with different polymers, good water solubility and good processibility. It is frequently incorporated into PLA,^[^ ^53^, ^54^ ^]^ cellulose acetate,^[^ ^36^, ^55^, ^56^ ^]^ polycaprolactone^[^ ^57^, ^58^ ^]^ etc. fibrous support matrix, especially in the biomedical field. However, few research has used high molecular weight PEG, also known as polyethylene oxide (PEO) as a support matrix due to the low melting point of PEO and poor mechanical strength. Nevertheless Faridi‐Majidi et al.^[^ ^39^ ^]^ managed to fabricate a PEO nano fiber matrix mixed directly with pure hexadecane (HD) and with nanoencapsulated hexadecane (NHD) as PCM components. SEM images showed good encapsulation of HD and NHD. Unfortunately, form‐stability was not reported and we are unable to evaluate the effectiveness of PEO as a fibrous matrix. Fredi et al.^[^ ^29^ ^]^ on the hand fabricated a form‐stable PEO fibrous mat by crosslinking the PEO chains with trimethylolpropane triacrylate crosslinker. With PEO as the PCM and matrix, its tensile strength and strain at break improved by 5 times after crosslinking, and was form‐stable with no weight loss when heated to 100°C for 2 min. These examples demonstrated that PEO is capable to be electrospun as a fibrous support matrix for PCMs, and PEG has also been successfully incorporated in electrospun fibers. The merger of these two same natured material would possibly provide favorable interactions for the formation of form‐stable PCM fibers.

To date, there has been no reports of PEG acting as both the matrix and PCM or plasticizer. The closest related work was conducted by Ke et al.^[^ ^59^ ^]^ where they electrospun a PAN fibrous mats loaded with a PEG 1000:PEG 2000 (1:1) binary mixture and attained form‐stability with a melting enthalpy of 182 J g^−1^. Another related work was carried out by Sun et al.^[^ ^60^ ^]^ where they fabricated a flexible form‐stable PCM polymer gel using PEG400/PEG2000/ sodium stearate (NaR). PEG400 in the above case remained as a liquid throughout the working temperature range (20–80°C) to allow for easy nucleation and polycrystalline growth. In addition, NaR provided a 3D network with micropores to contain the binary PCM mixture to prevent leakage. In both cases, a binary mixture of different molecular PEGs was used, but with a different chemical natured matrix to support their form‐stability and working temperature range. In this work, we report the use of a high molecular weight PEO (Mv 600,000) (PEO(600K)) physically mixed with a low molecular weight PEG (Mv 950–1050) (PEG(1050)) at different ratios, to electrospin into a fibrous mat and study its form‐stability, phase change properties and mechanical properties. The addition of PEG(1050) significantly improved the mechanical properties of the fibers, reduced its supercooling, and two‐phase transition segments were observed, with the latent heat for the PEG(1050) segment achieving up to 22 J g^−1^.

## 2 DISCUSSION

### 2.1 Morphological analysis

**FIGURE 1:**
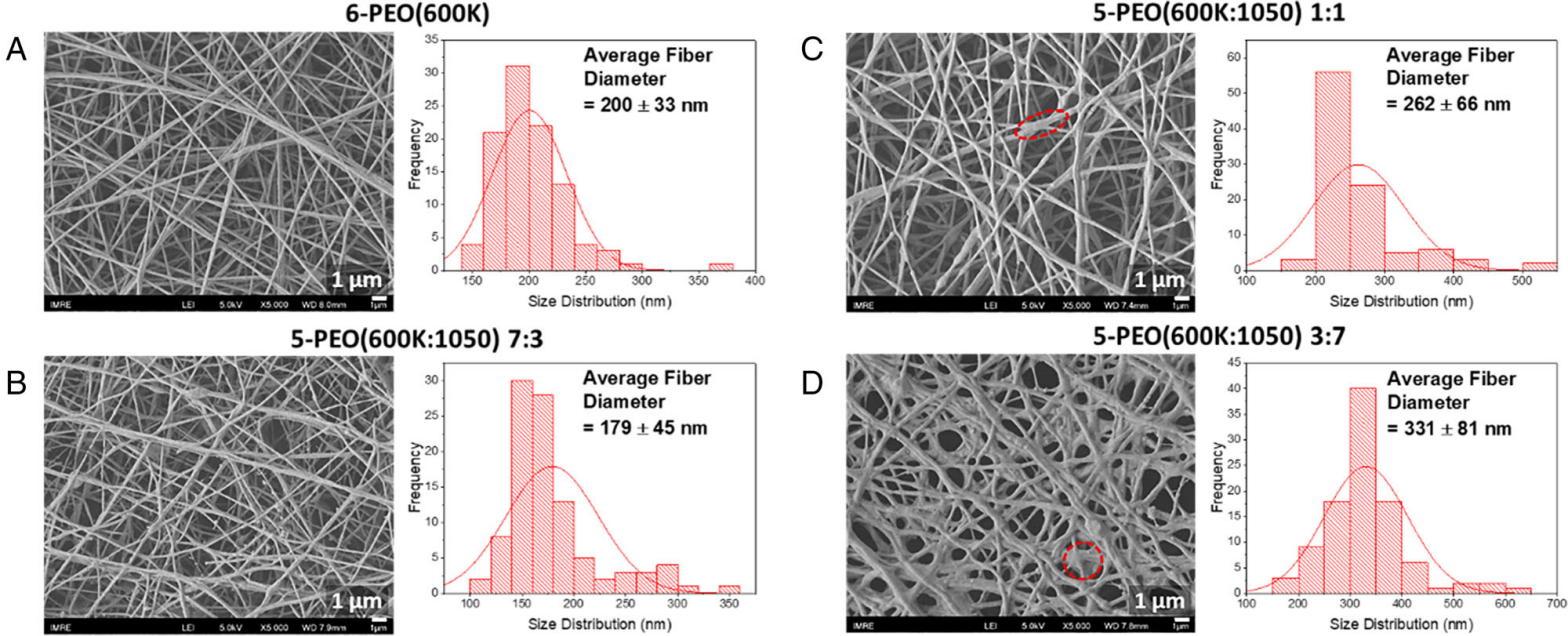
SEM analysis (scale bar: 1 μm) and its respective diameter distribution curve for (A) 6‐PEO(600K), and 5‐PEO(600K:1050) (B) 7:3, (C) 1:1, (D) 3:7. In the SEM image, a red dashed circle is used to highlight one of the adhesion structures present.

**FIGURE 2:**
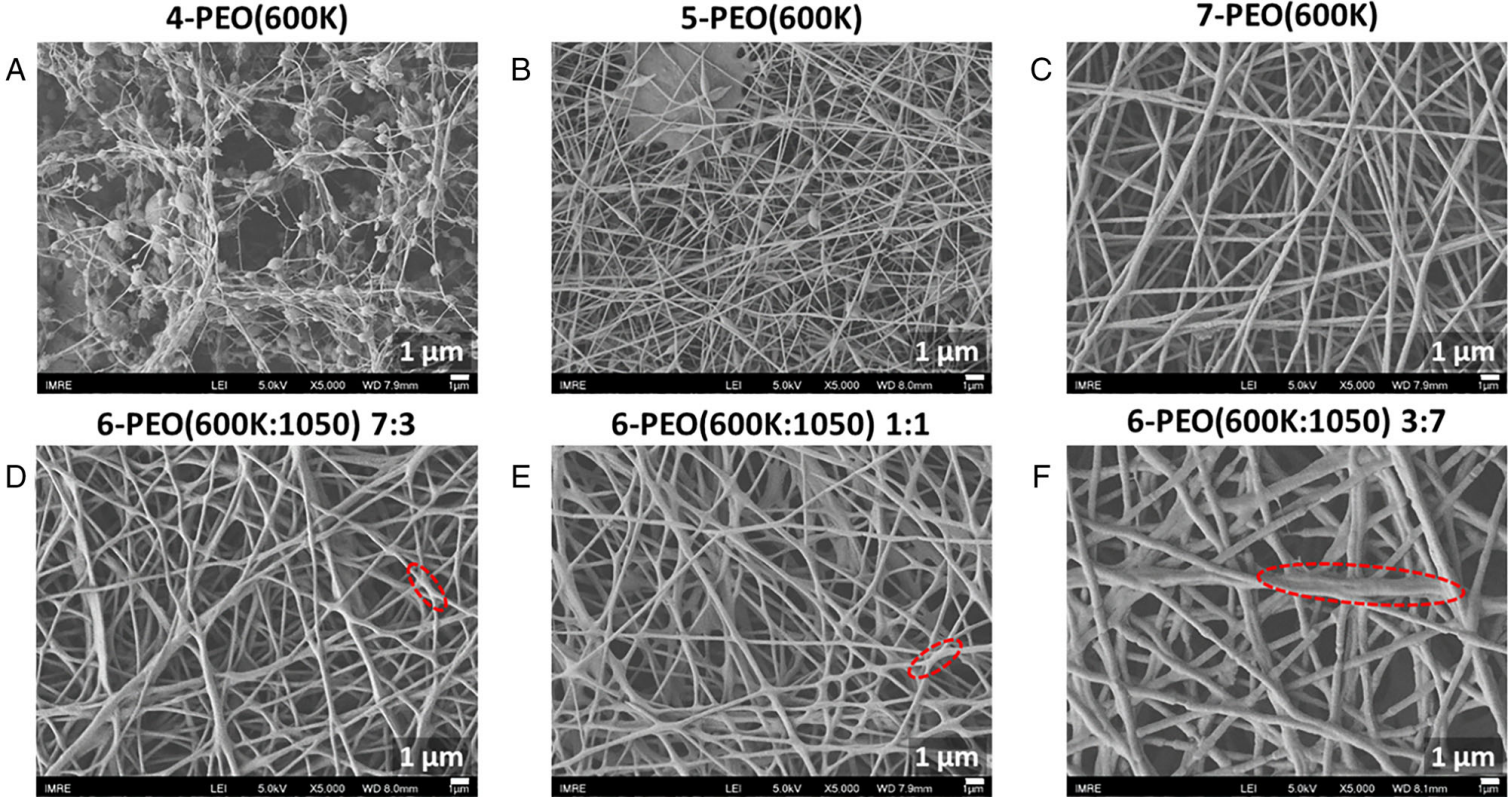
SEM analysis (scale bar is 1 μm) for (A) 4‐PEO(600K), (B) 5‐PEO(600K), (C) 7‐PEO(600K), and 6‐PEO(600K:1050) (D) 7:3, (E) 1:1, (F) 3:7, to study the optimal solution concentration for electrospinning. The red dashed circle highlights one of the adhesion structure present in the respective SEM image.

A series of 4–7 wt% X‐PEO(600K) solutions and 5–6 wt% X‐PEO(600K:1050) were electrospun according to a method as described in Section 2.2, and the microstructure of the fibers was studied using SEM (Figures 1 and 2). Excessive beading was observed for 4% and 5% PEO(600K) sample solutions, while the other samples with different concentrations showed comparable quality fibers.

Higher loading of PEG(1050) led to an increase in fiber diameter (Figure 1B‐D), and this is likely due to the overall increase in electrospinning solution concentration. Also, higher PEG(1050) loading increased the occurrence of adhesion structures at the fiber intersections, which led to a less uniform morphology with larger standard deviation in the fiber diameter. Severe adhesion can be seen especially in PEO(600K:1050) 3:7 fibers. This is due to strong hydrogen bonds present between the PEO, PEG and water in the system, which prevented the complete vaporization of the water solvent.^[^ ^40^ ^]^ No adhesion structures were observed for fibers spun by 5‐PEO(600K:1050) 7:3, and 6‐ and 7‐PEO(600K) solutions (Figures 1B,A and 2C), thus indicating that they are suitable for electrospinning.

On deciding the concentration of the X‐PEO(600K) and X‐PEO(600K:1050) solutions for electro‐spinning, two factors need to be taken into account. Firstly, an increase in the electrospinning solution's concentration by 1% greatly increased the viscosity, which made it more challenging to process. Secondly, the higher concentration solutions tend to produce more surface agglomerates after electrospinning for a long duration of about 30 h. This might be due to inefficient evaporation of the solvent (water), causing PEO to clump together. Additionally, for the composite fibers, minor leaching and accumulation of PEG(1050) on the surface may occur due to its melting point being closer to room temperature. Hence the optimum concentration for PEO(600K) and the PEO(600K:1050) solutions were determined to be 6% and 5%, respectively, and these samples were used for subsequent characterizations and tests.

### 2.2 Chemical structure analysis

**FIGURE 3:**
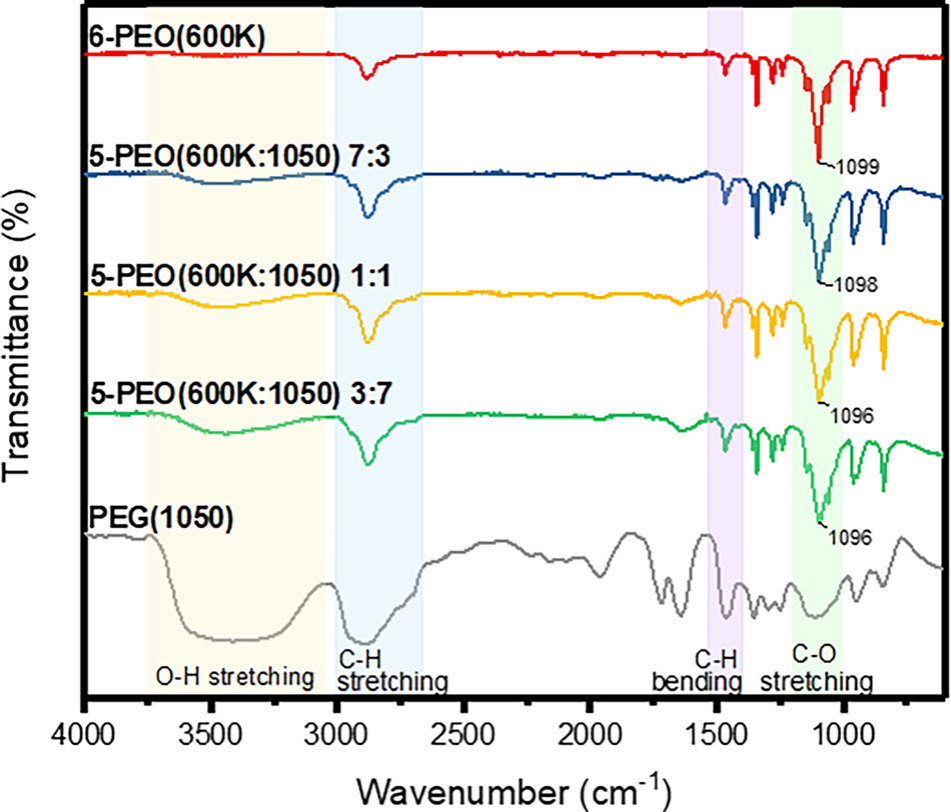
FTIR spectra for the electrospun samples and PEG(1050).

FTIR analysis was carried out on the respective electrospun fibers in the ATR mode, and on PEG(1050) in the transmission mode (Figure 3). The observed peaks are in good agreement with the structure of PEO and PEG. For example, the characteristic peaks around the region of 3470 cm^−1^ corresponds to the OH stretch of the terminal hydroxyl groups, 2886 cm^−1^ to the C─H stretching of the methyl group, 1467 cm^−1^ to C─H bending, and 1099 cm^−1^ to the C─O stretching. The other prominent peaks belong to vibrations of the methyl group, where 1342 cm^−1^ corresponds to CH_2_ wagging, 1280 cm^−1^ and 1242 cm^−1^ to C─H in‐plane bending, and 962 cm^−1^ and 842 cm^−1^ to C─H out‐of‐plane bending vibrations.^[^ ^61^, ^62^, ^63^ ^]^ Notably, the intensity of O─H band increased with PEG(1050) loading due to an increase in proportion of terminal hydroxyl group. Also, the triplet peak at around 1148 cm^−1^, 1099 cm^−1^ and 1061 cm^−1^ becomes less sharp and broader with PEG(1050) loading, and a broad band is observed for PEG(1050) without distinct peaks. The reduction in peak sharpness indicates a decrease in crystallinity,^[^ ^61^ ^]^ which is an indication that the presence of PEG(1050) acts as a plasticizer for PEO(600K).^[^ ^64^ ^]^ Also broadening of the peaks indicates an increase in hydrogen bonding, which is in line with the earlier suggestion of an increase in terminal hydroxyl groups. The peaks at 1099 cm^−1^ shifted to lower wavenumbers as PEG(1050) loading increased, indicating that hydrogen bonding possibly occurred^[^ ^65^ ^]^ between the ─OH group, and the ─CO group. PEO(600K‐1050) sample spectrums appear to be an overlap of both 6‐PEO(600K) and PEG(1050) spectra, indicating only the presence of physical interaction without forming chemical bonds.

### 2.3 Phase change properties and thermal stability

**FIGURE 4:**
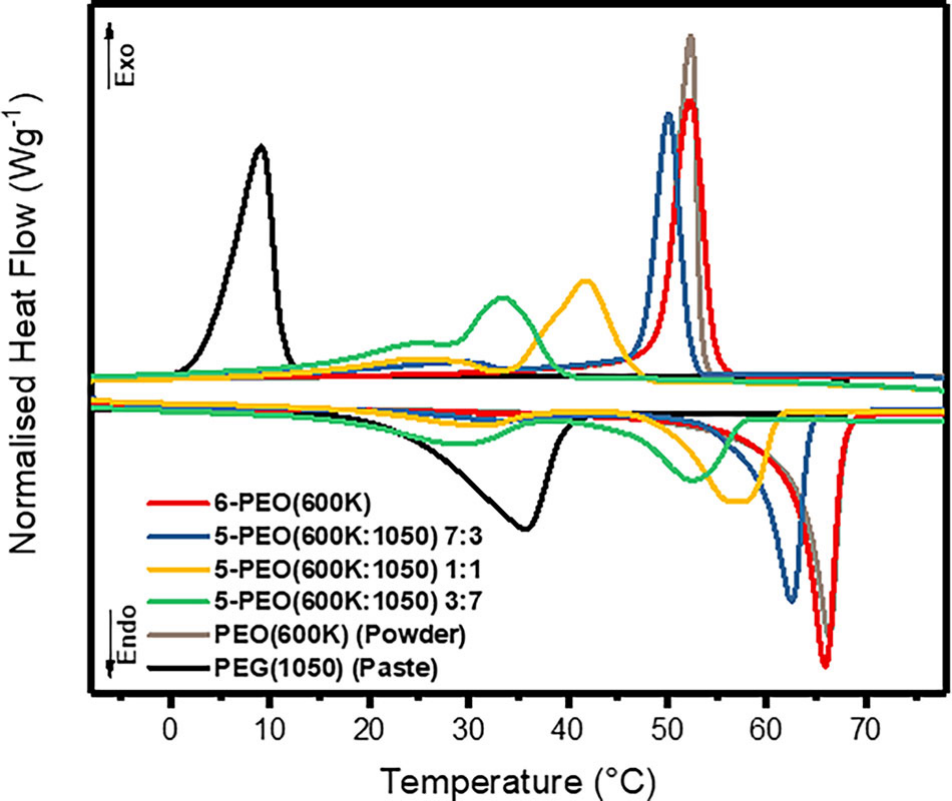
DSC curves for electrospun samples, PEO(600K) (Powder) and PEG(1050) (Paste), of which the latter two are the pure chemical form.

Phase change properties of electrospun samples were analyzed via DSC as shown in Figure 4 and Table 1. The Δ*H* and transition temperatures of electrospun 6‐PEO(600K) and its pure form PEO(600K) (Powder) were found to be similar, indicating that the process of electrospinning does not affect its phase change properties. In contrast, for the other electrospun PEO(600K:1050) samples, two peaks were observed in their melting and solidification cycles. The lower temperature peak was attributed to the PEG(1050) component, while the higher temperature peak was attributed to PEO(600K) component. *T_m_* and *T_c_* of both components were lower than the peak temperatures of its pure form, suggesting a cryoscopic effect as each component exists as an impurity in the other component's system.^[^ ^66^, ^67^ ^]^ But Δ*H_m_* of the PEO(600K) component was higher than the theoretical values by 10–40%, while Δ*H_m_* of the PEG(1050) component was significantly lower than its theoretical values by 61–75%. This could be understood based on the concept that *T_m_* is proportionate to the crystallite size while Δ*H_m_* depends on the number of crystallites.^[^ ^68^ ^]^

**TABLE 1:** DSC analysis data of electrospun samples, PEO(600K) (Powder) and PEG(1050) (Paste).

	PEG(1050)						PEO(600K)					
Sample	*T_m_*_, 1050_ [°C]	Δ*H_m_*_, 1050_ [J g^−1^]	*T_mo_*_, 1050_ [°C]	*T_c_*_, 1050_ [°C]	Δ*H_c_*_, 1050_ [J g^−1^]	*T_co_*_, 1050_ [°C]	*T_m_*_, 600 K_ [°C]	Δ*H_m_*_, 600 K_ [J g^−1^]	*T_mo_*_, 600 K_ [°C]	*T_c_*_, 600 K_ [°C]	Δ*H_c_*_, 600 K_ [J g^−1^]	*T_co_*_, 600 K_ [°C]
PEG1050 (Paste)	35.7	125.4	23.4	9.1	133.1	11.2	–	–	–	–	–	–
PEO(600K) (Powder)	–	–	–	–	–	–	66.3	125.0	62.9	52.3	120.7	53.3
6‐PEO(600K)	–	–	–	–	–	–	65.8	126.5	62.7	52.3	124.1	54.6
5‐PEO(600K:1050) 7:3	33.2	9.4	23.5	28.6	8.3	32.6	62.5	97.0	58.7	50.1	95.5	52.2
5‐PEO(600K:1050) 1:1	31.1	22.2	18.82	24.5	19.9	32.0	57.3	81.2	50.1	41.8	74.5	46.4
5‐PEO(600K:1050) 3:7	28.1	33.9	16.9	24.2	114.1^*^	38.9^*^	52.6	52.6	45.9	33.5	114.1^*^	38.9^*^

* Δ*H_c_* and *T_co_* values from combined peaks of PEG(1050) and PEO(600K).

Each component exists as a defect and reduces the crystallinity and size of crystallites of the other component.^[^ ^69^, ^70^ ^]^ Though they have the same structure, the significant difference in the molecular weight hinders uniform crystallization. In the case of the PEO(600K) component, increase in loading of PEG(1050) led to a reduction in crystallinity of PEO(600K), and thus a decrease in *T_m_* and *T_c_*. However, PEG(1050) also serves as crystallization sites to increase the number of crystallites of PEO(600K), which results in the Δ*H_m_*_, 600K_ higher than its theoretical values. Nevertheless, Δ*H_m_*_, 600K_ demonstrated a decreasing trend with increasing PEG(1050) loading as the PEG(600K) concentration decreased.

As for the PEG(1050) component, its molecular weight is much smaller than the matrix, thus it experienced a confinement effect,^[^ ^71^ ^]^ which is more prominent in samples with lower PEG(1050) loading. 5‐PEO(600K:1050) 7:3 experienced the largest deviation in Δ*H_m_* by 75%, where a large portion of the short chain PEG(1050) was confined within the PEO(600K) matrix, restricting proper crystallization of PEG(1050). As such its latent heat potential is not fully harnessed, resulting in a much smaller Δ*H_m_* compared to its theoretical value. As PEG(1050) loading increased, some PEG chains may transit into the partially confined state where part of the chain sticks out from PEO(600K) matrix. The sticking out portion can partially crystallize and form more tiny crystallites, resulting in an increase in Δ*H_m_*, but still lower than the theoretical values.

A reduction in supercooling for PEG(1050) component was also observed as a result of the confinement effect. The number of nucleation sites increased, which allowed nucleation to take place at a higher temperature. In addition, large crystal growth is restricted and faster transition between the different phases occurs. This sharply increased the *T_c_*_, 1050_ by 15–19°C and slightly decreased the *T_m_*_, 1050_ by 2.5–7.6°C, narrowing the supercooling from 26.7°C to 3.9–6.6°C which makes the fibers for thermally stable and functional. The combined cryoscopic and confinement effect on varying compositions of PEO/PEG fibers allowed for tuning of its working temperature, where increasing the PEG(1050) concentration would decrease the overall *T_m_*. As such, PEO:PEG fibers can be fine‐tuned to meet specific temperature requirements of potential applications. Nevertheless, the final formulation would still need to factor in the ratio limits for the fibers to remain form‐stable, which will be discussed in the next section.

**FIGURE 5:**
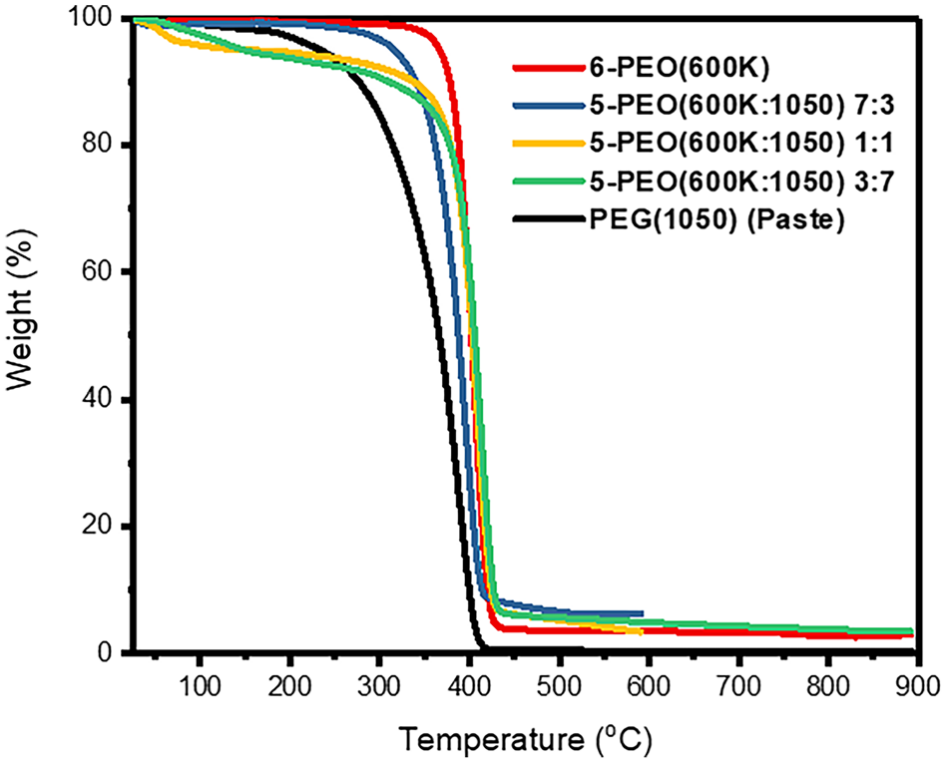
TGA analysis graphs for electrospun fibers and PEG(1050) (Paste), of which the latter is the pure chemical form.

TGA was performed on the electrospun fibers (Figure 5). The results show that 6‐PEO(600K) and 5‐PEO(600K:1050) 7:3 underwent a one‐step decomposition starting around 270–330°C, with peak degradation around 395–405°C. For 5‐PEO(600K:1050) 1:1 and 3:7, a small degradation step occurring between 40–160°C with rapid degradation around 60–90°C was observed, and weight loss amounted to 3.8% and 5.9%, respectively. These small degradation steps were attributed to the presence of moisture due to the hygroscopic nature of PEG,^[^ ^72^ ^]^ which would be more significantly absorbed by the higher amount of paste form PEG(1050). As such, 5‐PEO(600K:1050) 1:1 and 3:7 would also be thought to have good thermal stability similar to the other electrospun samples, making it functional in the working temperature range.

### 2.4 Form‐stability analysis

**FIGURE 6:**
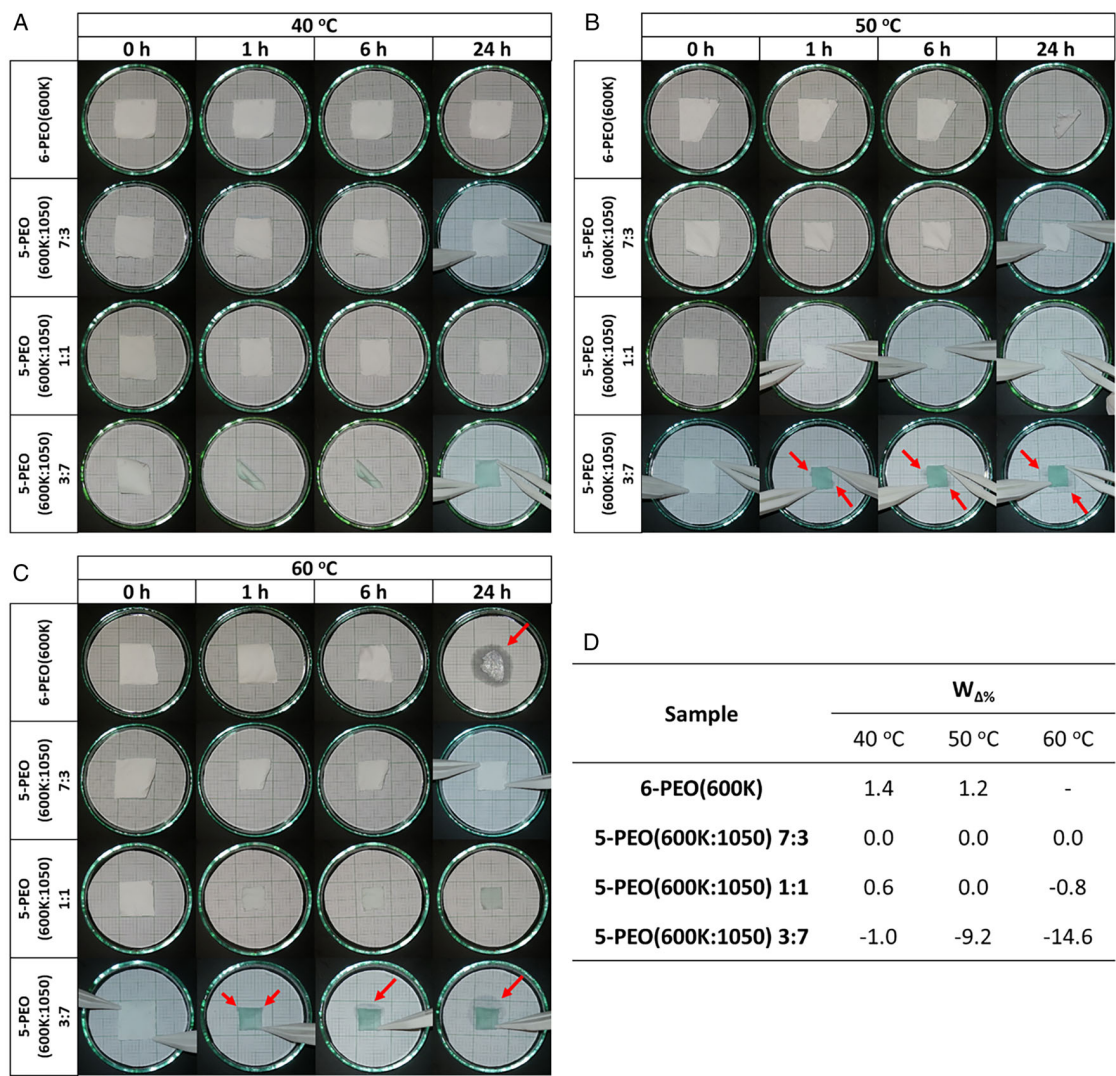
Form‐stability test of the electrospun fibers at (A) 40°C, (B) 50°C, and (C) 60°C, and (D) the percentage weight change (*W*_Δ%_) of each composite under the respective temperature at the end of the leakage test. Red arrows indicate leakage of PCM from the fibers.

**FIGURE 7:**
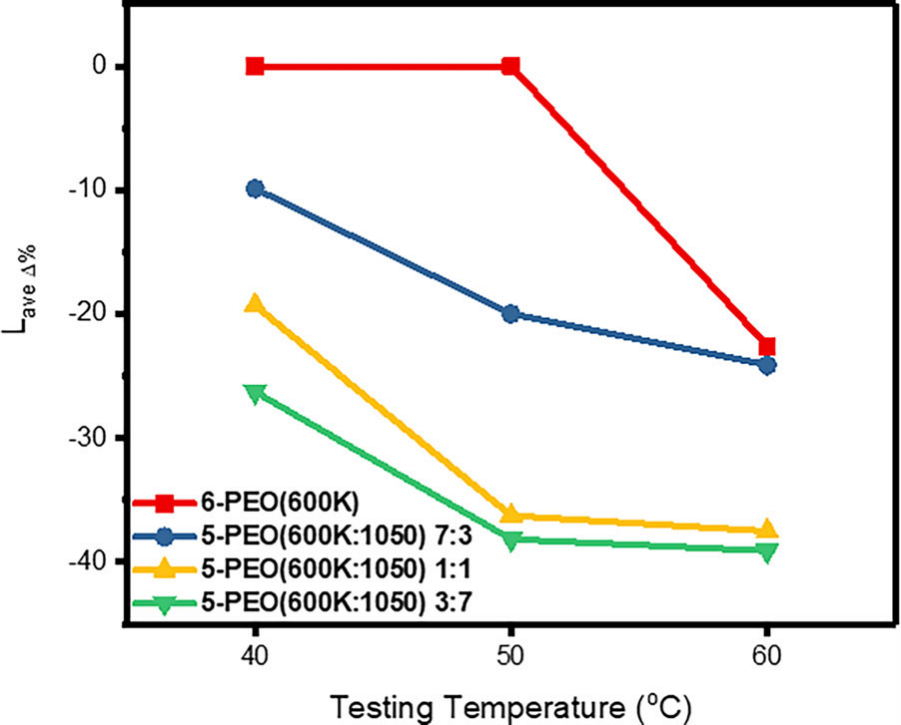
*L_ave_*_Δ%_ (average length) at each respective leakage testing temperature (Fiber dimensions of 6‐PEG(600K) were measured at 6 h, whereas the leakage test for the remaining samples were at 24 h).

**FIGURE 8:**
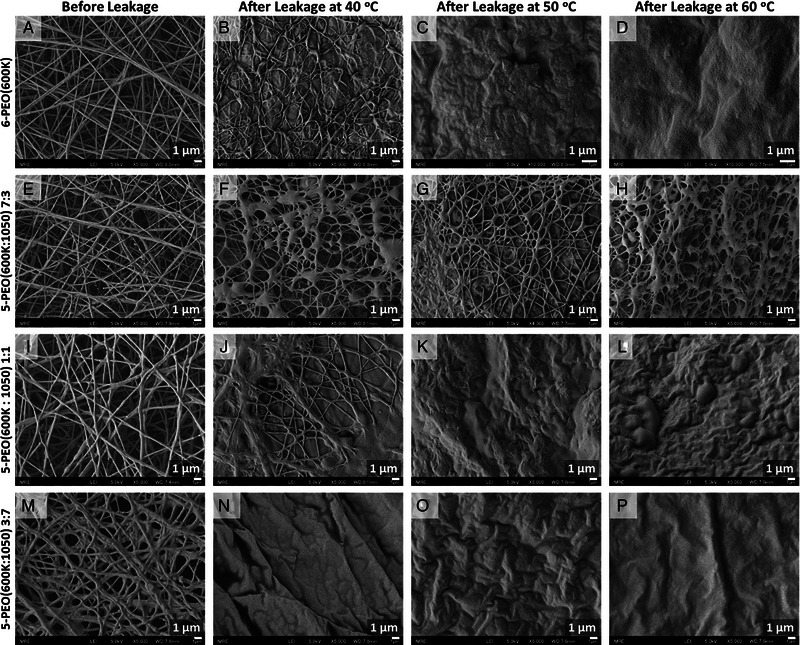
SEM images of PEO/PEG fibers before and after the leakage test conducted at the respective temperatures. SEM images of (A–D) 6‐PEO(600K), (E–H) 5‐PEO(600K:1050) 7:3, (I–L) 5‐PEO(600K:1050) 1:1 and (M–P) 5‐PEO(600K:1050) 3:7 fibers before and after leakage at 40°C, 50°C and 60°C respectively.

Form‐stability test was carried out as described in Section 4.3, and the leakage together with form/shape stability of electrospun samples were monitored at different temperatures (Figure 6). The results were compared by measuring *L_ave_*_Δ%_ of the samples (Figure 7). Following the leakage test, the fiber morphology was examined using SEM (Figure 8).

  

6‐PEG(600K) did not show visible shrinkage after being heated at 40°C for a prolonged time (Figure 6A). However, fibrous structures could still be observed under SEM, but with an increase in adhesion structures (Figure 8B). This could be due to presence of moisture in the sample or testing atmosphere, which, when under mild heat, caused the fibers to deform and stick together. In contrast, fibers remained visibly unchanged at 50°C for the first 6 h, but became crinkled after a prolonged heating time of 24 h (Figure 6B). This might be due to the slight elevation in temperature which causes greater movement/vibration to polymer chains, leading to the surface fiber morphology to completely breakdown (Figure 8C). However, since the temperature still has not reach its *T*_*mo*, 600K_ (62.7°C), the core fiber frame structure is possibly still intact, and partial crinkling of the fibers occurs only after prolong heating (6 h). When the fiber was tested at 60°C, it began to shrink slightly after 1 h, and after 24 h it crinkled up and appeared semi‐transparent (Figure 6C). When the testing temperature was close to the *T_mo_*_, 600K_ (62.7°C), PEO chain mobility is increased,^[^ ^73^ ^]^ leading to chain relaxation and reordering, subsequently causing the pores to collapse and result in shrinkage.^[^ ^74^ ^]^ As a result, partially collapsed matrix fills the pores between the fibers (Figure 8D), leading to reduced light scattering at fiber‐air interfaces, making the fiber semi‐transparent at the end of 24 h.

On the other hand, all PEG(1050) loaded fibers experienced shrinkage at the initial testing temperature of 40°C, and rising testing temperature and increasing PEG(1050) loading led to increase in shrinkage (Figure 6). This is because the fibers were tested at temperatures above the melting point of the PEG(1050) component of the sample. PEG(1050) therefore exists as a fluid phase and act as a plasticizer for the PEO(600K) polymer chains. Interacting with the plasticizer allowed the polymer chains to release built‐up stress and remain in a low energy state.^[^ ^75^ ^]^ As a result, mobility and free volume of the PEO(600K) matrix increased with reduced chain entanglements,^[^ ^76^, ^77^ ^]^ resulting in shrinkage of the macroscopic fiber structure. With a higher PEG(1050) loading, plasticizer content increased causing greater shrinkage correspondingly.

To compare the average shrinkage based on *L_ave_*_, Δ%_ values (Figure 7), 5‐PEO(600K/1050) 7:3 recorded a rather consistent decrease from 40 to 60°C due to its *T_m_*_, 600K_ (62.5°C) being above the testing temperatures. The PEO(600K) matrix does not melt completely, and fibrous networks could still be observed for all temperatures (Figure 8F,G,H). Its shrinkage thus increased with higher temperatures as the two PEG components become more miscible. On the other hand, 5‐PEO(600K/1050) 1:1 and 3:7 experienced similar drastic shrinkage when the testing temperatures were above their *T_mo_*_, 600K_ (50.1 and 45.9°C, respectively). At 50°C, the samples began to melt and the PEO(600K) support matrix underwent collapse, causing a sharp shrinkage. In fact, their PEO(600K) matrix began to partially collapse at 40°C as shown in the SEM images with featureless continuous medium and crinkled surface (Figure 8K,L,N‐P), except for 5‐PEO(600K:1050) 1:1 that still showed a small fibrous area at 40°C (Figure 8J). This is attributed to the additional miscible effect at a higher PEG(1050) loading. When the testing temperature increased to 60°C, both samples thus experienced a minimal increase in shrinkage as the bulk matrix has already collapsed. Nevertheless, all samples were able to maintain its square or rectangular shape at the end of 24 h for all temperatures without crinkling compared to pure PEO(600K) fibers. This is due to the presence of PEG(1050) acting as plasticizer which gave the PEO(600K) matrix higher mobility and thus control over the shrinkage, allowing it to maintain its rectangular shape instead of crumpling up into an irregular shape.

Despite shrinkage observed for the fibers, leakage was detected only for PEO(600K:1050) 3:7 with wet stains as indicated by the red arrows in Figure 6. The weight of the samples (those that can be removed from the graph paper after leakage) were calculated to estimate the percentage weight change (*W*_Δ%_) (Figure 6D). Only PEO(600K:1050) 3:7 exhibited a leakage at the testing temperature of 40°C mainly due to its high PEG(1050) loading as well as low *T_mo_*_, 600K_ (45.9°C). Leakage increased with increase in testing temperature for PEO(600K:1050) 3:7 which caused PEO(600K) matrix to collapse when the testing temperature eventually rose above *T_m_*_, 600K_. A minor leakage was also detected for PEO(600K:1050) 1:1 at 60°C due to the same reason. Interestingly, a few samples showed an increase in final weight, possibly due to the hygroscopic nature of PEO that absorbs moisture rapidly when taken out from the heated environment. All other samples were form‐stable with zero to minimal *W*_Δ%_ and PEO(600K:1050) 7:3 is the best performing film with least shrinkage and no leakage.

### 2.5 Mechanical properties

**FIGURE 9:**
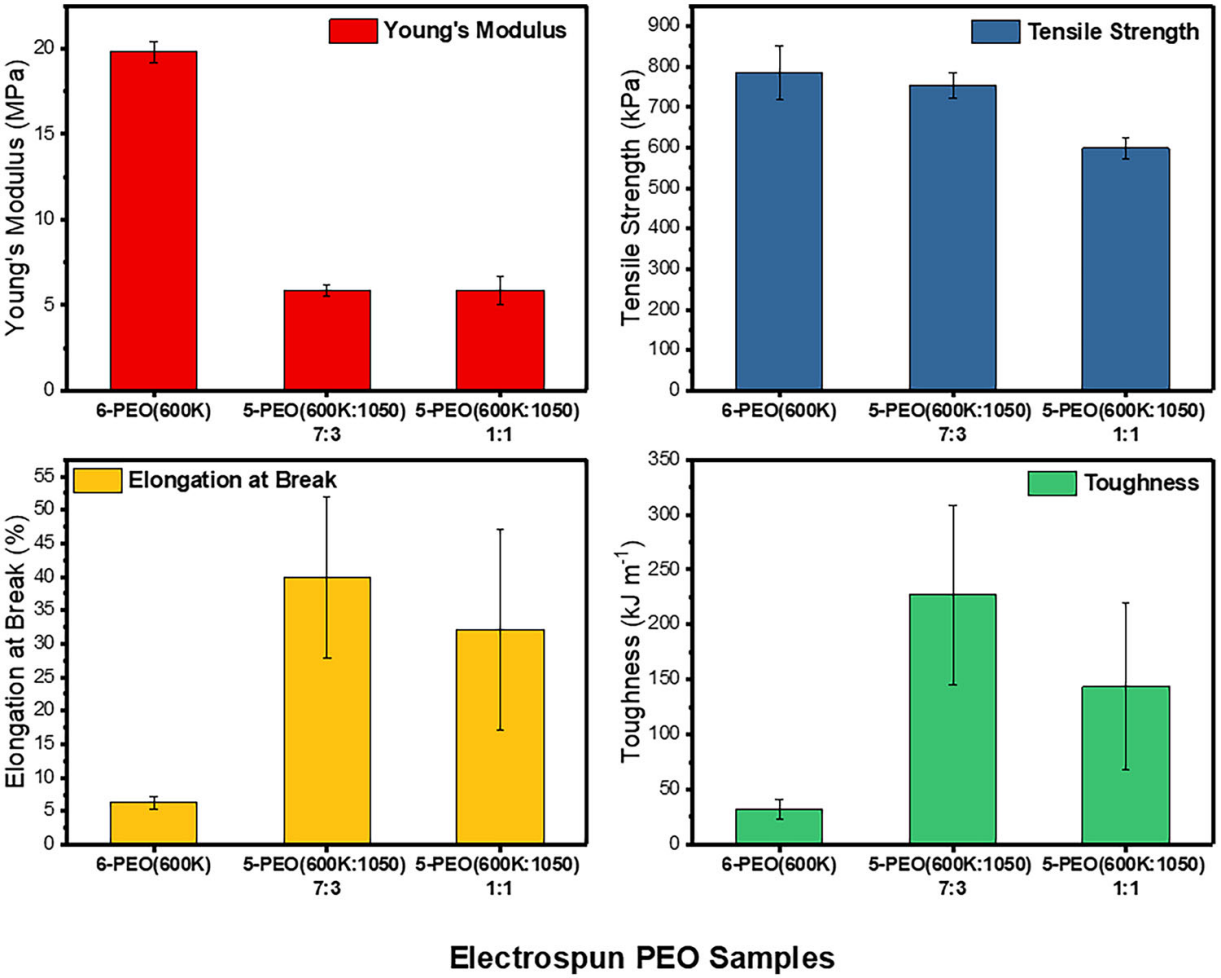
Mechanical properties including Young's modulus, tensile strength, elongation at break and toughness.

The mechanical properties of the three best fibers, 6‐PEO(600K), 5‐PEO(600K‐1050) 7:3 and 1:1 are summarized in Figure 9. 6‐PEO(600K) gave a Young's Modulus of 19.81 MPa, which is about 3.4 times higher than 5‐PEO(600K‐1050) 7:3 and 1:1 fibers, due to the absence of PEG(1050) as plasticizer in its fibers. Consequently, the composite 5‐PEO(600K‐1050) fibers have a significantly larger elongation at break than 6‐PEO(600K) (6.29%) by 6.4 and 5.1 times respectively, confirming the improvement in elastic property of the fibers. Though the composite fibers have a greater toughness than 6‐PEO(600K) (32.45 kJ m^−1^) by 7 and 4.4 times respectively, they have a lower tensile strength (785.44 kPa) by 4% and 24%, respectively. This could be due to the lower amount of PEO(600K) in the composites as supporting matrix, resulting in an overall weaker structure. It is interesting to note that 5‐PEO(600K‐1050) 7:3 has a larger elongation at break and toughness than 5‐PEO(600K‐1050) 1:1, despite the latter having a higher amount of plasticizer to provide fluidity. A possible reason is that the increase in fluidity was compromised by the lack of structural matrix support from a lower amount of PEO(600K) as indicated by the latter's lower tensile strength. Hence 5‐PEO(600K‐1050) 7:3 has the best mechanical properties of flexibility, strength and durability.

**FIGURE 10:**
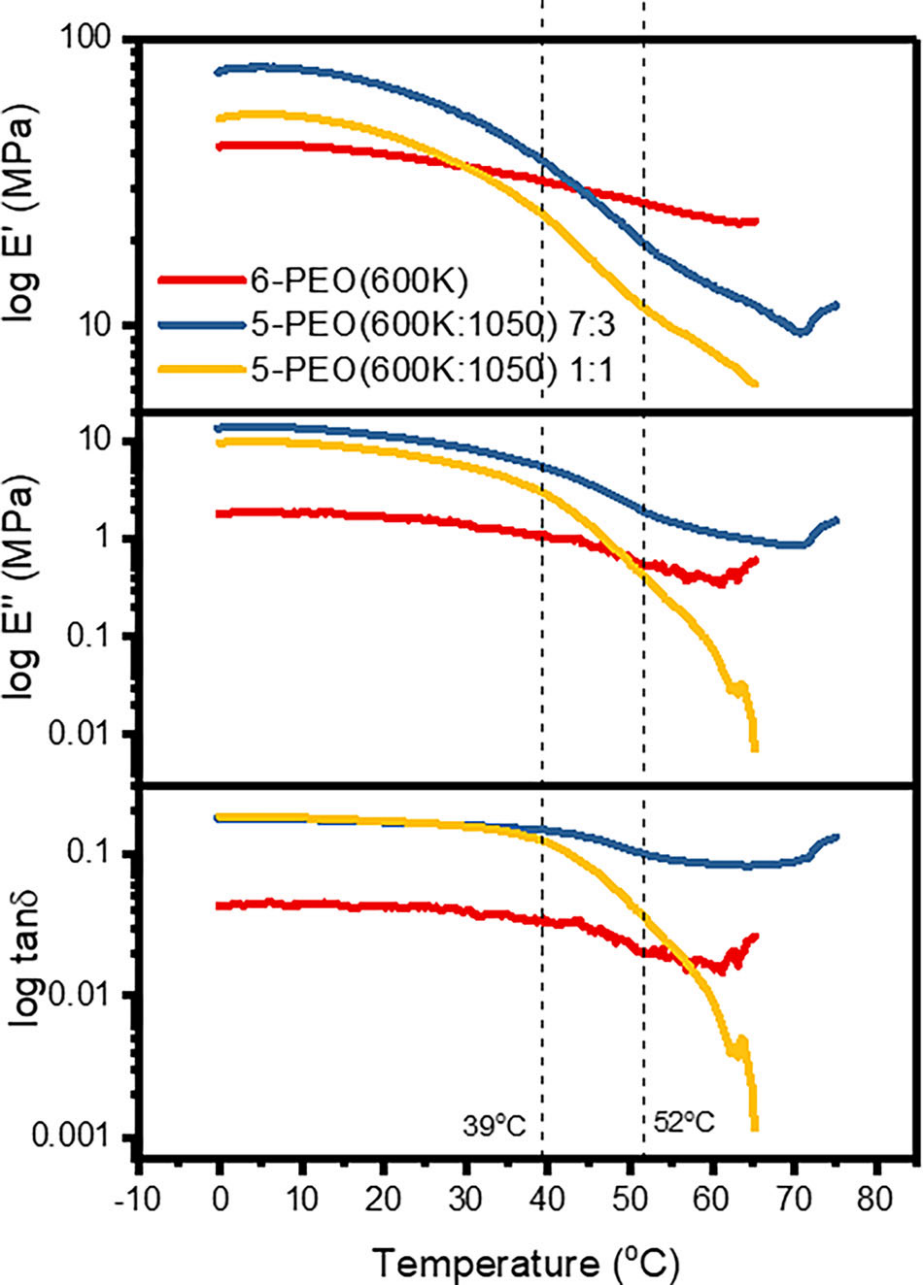
DMA analysis for storage modulus (*E*′), loss modulus (*E*″), and loss factor (tan*δ*) with respect to temperature.

DMA was conducted to analyze the thermomechanical behavior of the same three fibers, and their storage modulus (*E*′), loss modulus (*E*″), and loss factor (tan*δ*) were compared with each other (Figure 10). 6‐PEO(600K) has the lowest *E*′ due to the absence of PEG(1050) to hinder PEO(600K) chain motion. The lower *E*′ for 5‐PEO(600K:1050) 1:1 compared to its 7:3 counterpart was due to increasing “soft” PEG(1050) component in the fibers to offer higher flexibility. When the composite fibers were heated, the onset of the decreasing *E*′ value was observed between 16–19°C, followed by a steep drop between 39–52°C which corresponded to the melting phase of the PEG(1050) component. Therefore, this region defines the plasticization region where liquid PEG(1050) becomes increasingly miscible with the PEO(600K) matrix, causing *E*′ and *E*″ to decrease. With further increase in temperature, 5‐PEO(600K:1050) 1:1 sample breaks around 65°C during the test, suggesting an overall weakened structure due to a lower PEG(600K) matrix content. While PEO(600K:1050) 7:3 sample was able to remain intact till 80°C, agreeing well with the mechanical test results that it possessed better strength and durability. As for 6‐PEO(600K), the drop in *E*′ appears to be much gentler than the composite fibers as it exists as a single component. Eventually, *E*′ of 5‐PEO(600K:1050) 7:3 and 1:1 drops below that of 6‐PEO(600K) at 44°C and 30°C due to the plasticization effect. Thus, 5‐PEO(600K:1050) 7:3 gives the best thermomechanical performance.

## 3 CONCLUSION

PEG is widely used as a PCM due to its high latent heat and wide working temperature range. Also with its biocompatibility and non‐toxicity, it is widely applicable in the biomedical field for wound dressings and controlled drug delivery. As such, a flexible PEG based material is ideal for applications. Since PEG is unable to be drawn into fibers to fabricate the flexible component, a support matrix of the same nature, PEO is used. In this work, we have successfully electrospun PEG and PEO into fibrous mats, which demonstrated good compatibility and produced uniform fibers. A good latent heat of up to 33.9 J g^−1^ was achieved, with working temperatures between 28–33°C. In fact, working temperatures of these PEO/PEG fibers can be fine‐tuned by appropriately adjusting the composition of PEO:PEG, while taking into account of the composition limits for fibers to maintain form‐stability. The composite fibers exhibited a clear superiority in mechanical and thermomechanical strength as opposed to pure PEO(600K) fibers due to presence of PEG(1050) conferring plasticization effect. 5‐PEO(600K:1050) 7:3 gave the best performance, demonstrating good chemical and form‐stability, mechanical strength and least shrinkage. The flexibility and thermoregulatory function of the PEO/PEG fibers makes it useful and applicable in different sectors, including the biomedical field for thermoregulating or thermal responsive wound dressings; and thermoregulating fabrics for clothings, curtains, bags. Also, in light of the growing trend towards electronic wearables, the flexible PCM can be molded to take on the contour of the wearable device and improve contact between the device components. In addition, a PCM‐based fibrous mat could be used to make facemasks to provide thermal comfort to wearers, especially during a pandemic when wearing mask is made compulsory.

This study showcased the potential of PEO/PEG fibers as a flexible PCM fibrous mat with potential applications in different field. As this work is in its exploratory phase, future exploration direction can be geared towards enhancing the fiber properties or worked towards adding new functions in the fibers to cater to specific industries. Some exploratory directions include: (1) Improving mechanical strength—for the fibrous mats to be used in real applications, mechanical strength of the existing fibers need to be increased. Crosslinking agents, nanofillers, fiber reinforcements etc. can be added and electrospun into fibers to strengthen the fibers. Different polymer blends with PEO can also be explored to harness the advantages of each polymer and find a compatible system with the desired mechanical properties. (2) Enhancing flexibility—most applications such as fabrics, masks, bandages etc. require a flexible substrate that does not break when bent or wrapped around objects. Different types of plasticizers such as triethyl citrate, ethylene carbonate, dibutyl phthalate etc., on top of the PCM component added, can be investigated to find a compatible and optimum formulation for flexible fibrous substrates. (3) Optimizing phase change properties—tuning the ratio of PEO:PEG to vary the working temperature has a small temperature coverage, and any changes in the ratio may affect form‐stability or suitability in electrospinning. Therefore, different PCMs with melting point within the required application's temperature range can be used in the fiber system and tested for compatibility and workability. This would allow the fibers to extend their working temperature range which makes it more versatile. Latent heat will therefore not be compromised if a desired temperature requires less of the PCM component to be added. Suitable PCMs with higher latent heat can also be chosen. Similar to this study, the new PCM and plasticizer to be studied may be selected as the same material. (4) Conductivity—applications in the electronics sector or devices may require conducting fibers, where conducting nanoparticles such as copper, silver, carbon nanotubes etc. can be added and electrospun out with the fibers. These conducting particles may also take on a dual role of nanofillers to improve the fiber's mechanical strength, and the ratio can be fine‐tuned to the desired levels. (5) Sustainable materials—currently PEO and PEG used in our fibers are both considered biodegradable and thus sustainable. In view of the new materials that will be added to the system to improve its properties, they can be geared towards using biodegradable materials to generate an eco‐friendly fibrous mat. Flexible PCM fibers opens up many different exploratory directions. When the fibers are optimally fine‐tuned, it would be highly useful in various applications.

## 4 METHODS

### 4.1 Materials and instrumentation

Poly(ethylene oxide) (Mv 600,000) (PEO(600K)) and polyethylene glycol (Mv 950–1,050) (PEG(1050)) were purchased from Sigma‐Aldrich. All chemicals were used as received without further purification.

#### 4.1.1 Preparation of electrospinning sample solutions

X‐PEO (600K) electrospinning solutions were prepared by dissolving *X* wt% of PEO(600K) in deionized water. X‐PEO (600K:1050) solutions were obtained by adding PEG(1050) at different PEO (600K):PEG (1050) mass ratios of 7:3, 1:1 and 3:7 in deionized water. The respective mixtures were stirred overnight at room temperature until all solids dissolved. Prior to electrospinning, the mixture was sonicated for 30 min, and then degassed. Solution concentration was varied to identify the optimum formulation to produce uniform fibers for each sample ratio.

#### 4.1.2 Electrospinning

Sample solutions were electrospun via a nanofiber electrospinning system (NANON‐01A, MECC Co., Ltd., Japan). The respective sample solution was poured into a 6 mL plastic syringe attached with a stainless‐steel flat‐tipped 22G needle and kept at a distance of 15 cm between needle tips to a flat collector plate (20 × 15 cm). The solution was pumped out at a feed rate of 0.2 mL h^−1^, with an applied voltage of 10 kV, and spinneret speed was set at 20 mm s^−1^. Depending on the target sample size, spinneret width was set between 20 and 60 mm and electrospun between 2 and 30 h. The temperature was between 22 and 25°C, and relative humidity was between 55% and 65% during the electrospinning process. Electrospun samples were dried under vacuum at room temperature for 12 h before proceeding with other characterizations.

### 4.2 Form stability and leakage test

Electrospun fibers were cut into rectangular/square fragments of 2 × 2 (±0.5) cm, and the initial weight (*W_i_*), length of top (*a_i_*) and left (*b_i_*) edges were recorded. The respective fragments were then placed on a graph paper and inserted into a petri‐dish with a cover. Leakage test was then conducted at 40°C, 50°C and 60°C for 24 h. During the leakage test, samples were taken out from their test environment at regular timed intervals for image capture and form‐stability assessment, and returned back to the oven immediately. At the end of the leakage test, samples were allowed to cool down to room temperature, and the final weight (*W_f_*), length of top (*a_f_*) and left (*b_f_*) edges were recorded. The average length (*L_ave_*) is used to compare the overall change in fiber dimensions, where

(1a) $$L_{ave\;i}=\;\frac{\left(a_{i}-b_{i}\right)}{2}$$

(1b) $$L_{ave\;f}=\;\frac{\left(a_{f}-b_{f}\right)}{2}$$

Percentage change in weight (*W*_Δ%_) and average length *(L_ave_*_Δ%_) can be calculated by the following formula:

(2) $$X_{\Delta \%}=\;\frac{X_{f}-X_{i}}{X_{i}}\;\cdot 100$$

where *X* = *W* or *L_ave_*.

### 4.3 Characterizations

Surface morphology and fiber structure of samples being gold‐sputtered were analyzed by scanning electron microscopy (SEM) (JSM‐6700F, JEOL) using a field‐emission scanning electron microscope at a voltage of 5 kV and a current of 10 mA. Chemical structure for the fiber films were studied by attenuated total reflection Fourier transform infrared (ATR‐FTIR) spectrometry while PEG(1050) solid sample was analyzed under the transmission mode (Spectrum 2000, PerkinElmer) in a frequency range of 4000 to 600 cm^−1^, with a resolution of 4 cm^−1^ for 16 scans. Thermal stability was evaluated via thermogravimetric analysis (TGA) (Q500, TA Instruments) under a nitrogen flow of 60 mL min^−1^, at a heating rate of 20°C min^−1^ from room temperature to 600–900°C. Phase change properties were analyzed via differential scanning calorimetry (DSC) (Q100, TA Instruments) under 50 mL min^−1^ nitrogen gas flow rate, and at a rate of 10°C min^−1^ between −20°C to 80°C. Viscoelastic properties *E′*, *E″* and tan*δ* were captured via dynamic mechanical analysis (DMA) (Q800, TA Instruments) via a tension film clamp with the oscillation amplitude of 20 μm at frequency of 1 Hz, at a rate of 5°C min^−1^ from 0°C to 70°C. Mechanical properties were studied via Instron 5569 Table Universal testing machine with a load cell of 10 N at a crosshead speed of 2 mm min^−1^ and a gauge length of approximately 20 mm. Samples were prepared in rectangular stripe of 50 mm × 5 mm with a thickness of 0.1–0.4 mm.

## CONFLICT OF INTEREST STATEMENT

The authors declare no conflicts of interest.

## Data Availability

All data of this work are present in the article. The other data that support the findings of this work are available from the corresponding author upon reasonable request.
